# Heightened Responses of the Parahippocampal and Retrosplenial Cortices during Contextualized Recognition of Congruent Objects

**DOI:** 10.3389/fnbeh.2017.00232

**Published:** 2017-12-14

**Authors:** Daina Crafa, Colin Hawco, Mathieu B. Brodeur

**Affiliations:** ^1^Integrated Program in Neuroscience, McGill University, Montreal, QC, Canada; ^2^Campbell Family Mental Health Institute, Centre for Addiction and Mental Health, Toronto, ON, Canada; ^3^Department of Psychiatry, Douglas Mental Health University Institute, McGill University, Montreal, QC, Canada

**Keywords:** fMRI, scene context, object recognition, parahippocampal cortex, retrosplenial cortex

## Abstract

Context sometimes helps make objects more recognizable. Previous studies using functional magnetic resonance imaging (fMRI) have examined regional neural activity when objects have strong or weak associations with their contexts. Such studies have demonstrated that activity in the parahippocampal cortex (PHC) generally corresponds with strong associations between objects and their spatial contexts while retrosplenial cortex (RSC) activity is linked with episodic memory. However these studies investigated objects viewed in associated contexts, but the direct influence of scene on the perception of visual objects has not been widely investigated. We hypothesized that the PHC and RSC may only be engaged for congruent contexts in which the object could typically be found but not for neutral contexts. While in an fMRI scanner, 15 participants rated the recognizability of 152 photographic images of objects, presented within congruent and incongruent contexts. Regions of interest were created to examine PHC and RSC activity using a hypothesis-driven approach. Exploratory analyses were also performed to identify other regional activity. In line with previous studies, PHC and RSC activity emerged when objects were viewed in congruent contexts. Activity in the RSC, inferior parietal lobe (IPL) and fusiform gyrus also emerged. These findings indicate that different brain regions are employed when objects are meaningfully contextualized.

## Introduction

Objects in daily life are not always immediately recognized or known to the viewer, but they generally are easier to identify when the contextual information surrounding them is taken into account. The influence of contextual information depends on how strong the relationship is between object and context, and closer relationships increase the likelihood that the context will contribute to the recognition of the objects (Davenport and Potter, 2004). Functional magnetic resonance imaging (fMRI) studies examining relationships between object and context report that objects having strong associations with specific scene contexts (e.g., a tennis ball with a tennis court) elicit different brain activity than objects with weak or nonspecific associations (e.g., a generic rubber ball is associated with sports more broadly; Bar and Aminoff, 2003; Bar et al., 2005; Aminoff et al., 2007; Kim and Biederman, 2011).

Such studies have identified two cortical areas that together form parts of a larger neural network and play complementary roles in processing contextual associations between scenes and objects: the parahippocampal (PHC) and the retrosplenial (RSC) cortices. Higher activity in the PHC is mostly observed when objects with strong scene context associations are viewed; accordingly, the PHC appears to process place-related contextual information for objects with strong associations (Bar and Aminoff, 2003; Gronau et al., 2008; Kveraga et al., 2010). Furthermore, one study shows that even non-spatial scene associations congruently engage the PHC, which may suggest that the PHC is engaged with context processing more generally (Aminoff et al., 2007). A few studies have reported that the RSC also shows heighted activity for objects with strong associations and point to broader neuroscience literature linking RSC activity with episodic memory; thus, theorizing that the RSC accesses past memories when evaluating contextual associations (Bar and Aminoff, 2003; Vann et al., 2009). Numerous animal studies have investigated RSC activity during context processing (Keene and Bucci, 2008, 2009; Robinson et al., 2011). However, the relationships between RSC activity and context have not been directly examined in humans.

The interpretive influence of scene contexts on the recognition of objects does not only depend on the strength of their relationship, but it also depends on whether contexts have congruent associations with the object and how contextual information is applied to object recognition. Congruent context-object relationships refer to objects that typically appear in a given scene, whereas incongruent relationships refer to scenes in which the object appears odd or inappropriate. Several studies have shown that objects presented in congruent scenes are easier to recognize than objects placed in incongruent scenes or no scene at all (Oliva and Torralba, 2007). The context is more critical when the target object is difficult to recognize on its own (Klink et al., 2012; Dyck and Brodeur, 2015), such as when an object is unfamiliar, viewed from an unusual point of view, or when visibility is compromised by light and distance. In these circumstances, objects are visually ambiguous and should activate brain regions mediating the object-scene relationship, namely the PHC and RSC, when they are seen in a congruent scene (Rémy et al., 2014). While previous research has established that the PHC and RSC are activated by the object-scene associations that are initiated during perception of focal objects, which are centrally located in the visual field and presented in isolation, they have not generally examined whether these regions are involved when object recognition is viewed *within* a scene context.

The influence of scene context on object recognition brain processes has been mostly studied with event-related potentials (ERPs). These studies have shown that the electrophysiological brain activities of object recognition depend on how well the objects are congruent with the scene in which they appear (Ganis and Kutas, 2003; Mudrik et al., 2010, 2014; Demiral et al., 2012; Võ and Wolfe, 2013). Objects presented in incongruent scenes resemble the N400 response, which has been previously associated with incongruity, and elicit N390 and other negative ERPs over the central-frontal electrodes. To control for incongruity, a recent study from our lab investigated the effects of congruent contexts on ambiguous and unambiguous objects compared to a neutral condition in which presented objects neither appeared well-suited nor ill-suited (Dyck and Brodeur, 2015). The beneficial influence of congruent scenes was found on electrodes covering fronto-central and posterior areas. This study also demonstrated that ERPs of ambiguous objects viewed in congruent contexts resemble ERPs of unambiguous objects, meaning that the context effect mostly operates by reducing the ambiguity of percepts.

To our knowledge, Kirk (2008) has conducted the only fMRI study that directly investigated the influence of scene on the perception of visual objects. Their subjects made aesthetic judgments of stimuli including object in congruent scenes and objects in incongruent scenes. Greater activities for congruent stimuli were found in the lateral occipital cortex, the inferior parietal lobule, and the PHC. When the relationship between the object and the scene was unexpected, the frontal and parietal cortices, in addition to the RSC, were activated. In a subsequent study, Kirk et al. (2009) reported that frontal activity was related to a bias of judgment induced by the context. While these results show that the PHC and RSC activity are influenced by object-scene congruity, they showed that other brain areas, including the frontal and the parietal areas, are involved as well.

Building on previous research from our group, a study was conducted to evaluate brain activities during correct recognition of objects appearing in incongruent and neutral scenes. In contrast with Kirk (2008), subjects were instructed to try to recognize the objects and scenes were displayed before the target objects appeared. This mode of presentation is known to affect brain activity (Mudrik et al., 2010, 2014), likely because the scene has already been processed when the target object appears. It was hypothesized that PHC and RSC activity would correspond with congruent but not neutral contexts. Given the possibility of finding context-related activities in other regions of the brain, including the frontal and parietal areas, exploratory analyses were also conducted.

## Materials and Methods

### Participants

Fifteen healthy adult volunteers (6 females) between the ages of 18–27 (mean = 20.8, SD: 2.8) were recruited by placing ads on Kijiji and Craigslist. All subjects were right handed with normal or corrected-to-normal vision and showed no color blindness as verified with the Pseudo Isochromatic Plates Ishihara Compatible Test (Waggoner, 2010). Subjects reported no diagnoses of psychiatric or neurological disorders. The study was carried out and approved by the Research Ethics Boards of the Montreal Neurological Institute and of the Douglas Mental Health Institute, and all volunteers signed informed consent to participate in the experiment. All subjects gave written informed consent in accordance with the Declaration of Helsinki.

### Stimuli

**Figure 1:**
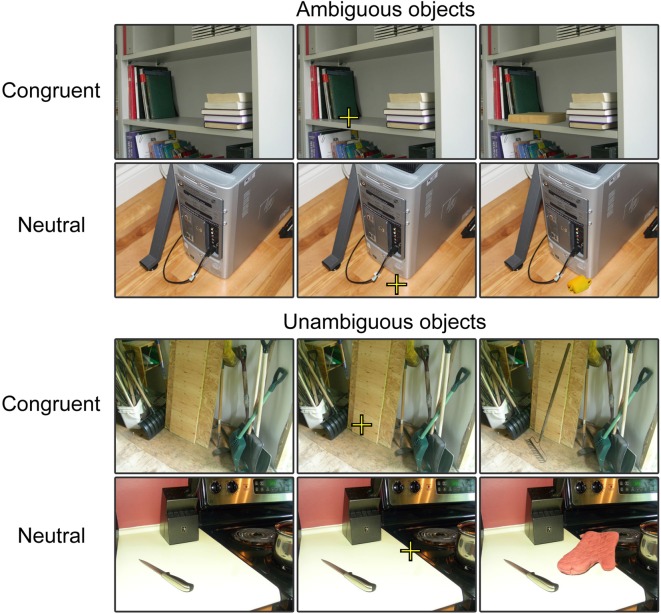
Examples of trials in each experimental condition. For the purpose of our experimental presentation, three versions of each stimulus were presented. First, the scene was presented without the object, second the scene was presented with the fixation embedded and third the scene was presented with the object embedded in it. These stimuli were presented sequentially at each trial to provide participants with sufficient time to process the scene, to locate the focal point and then to process the object within the scene.

Stimuli included 308 photographic images of objects, taken from the Bank of Standardized Stimuli (BOSS; Brodeur et al., 2010, 2014) such as those illustrated in Figure 1. Additional details regarding stimuli selection can be found in Dyck and Brodeur (2015). The objects recognizability was measured prior to the experiment. A pilot group consisting of 15 volunteers rated the recognizability of each target object presented alone, without scene, on a 3-point scale (0 = unrecognizable, 1 = had a vague idea, 2 = recognizable). The mean score for each object was computed. The mean score of object recognizability was 1.13 but there was a wide range of scores (SD: 0.70), meaning that some objects were easier to recognize than others.

Half of the objects were associated with *congruent scenes* and the other half, with *neutral scenes*. Congruent scenes are those in which the object could typically be found and neutral scenes are those that do not help with object recognition. Examples of scenes are presented in Figure 1. Congruity of context was validated by a different group of 15 volunteers who rated the images following the same procedure used to evaluate the recognizability of the objects except that this time, the objects were presented in their scenes. The mean score of objects in neutral scenes was 1.19 (SD: 0.21). This was just 0.05 over the score obtained when these objects were presented alone, over a blank background. In turn, the mean score of objects presented in congruent scenes was 1.82 (SD: 0.30). This represents an increase of 0.71 in comparison to when objects were presented alone. The mean score of object recognizability was significantly higher for congruent scenes, *t*_(306)_ = 47.82, *p* < 0.001.

### fMRI Scanning Procedure

Scanning was carried out on a 3.0T Siemens TimTrio System. Participants’ heads were stabilized using a vacuum cushion. Stimuli were presented using E-prime software 2.0 (Psychology Software Tools, Inc.) running on an IBM laptop computer, and an LCD projector and mirror system were used to display the images. Each trial started with a blank screen presented for 1 s followed by a scene. After 1 s, a fixation cross appeared on the scene at the target location and was replaced after 1 s by the target object, also for 1 s. Participants were asked to indicate how certain they were that they recognized the object by pressing one of three different buttons on a MRI compatible response pad. They pressed one button when they could not recognize the objects, another button when they had a vague idea of the object’s identity and on a third button when they recognized the object. Each trial was followed by a 2 s inter-stimulus interval (ISI). Participants completed a short eight trial practice before beginning the experiment in the scanner. Two runs of Echo-planar images (TR = 2000 ms, TE = 30 ms, flip angle = 90, 36 slices of 4 mm thick, 64 × 64 voxel plane with a FOV of 256 mm, giving 4 mm × 4 mm × 4 mm voxel size). Four volumes were discarded before each BOLD run, and MPRAGE was used for the anatomical scan (TR = 2300 ms, TE = 2.98 ms, FOV = 256 mm, 1 mm × 1 mm × 1 mm voxels, flip angle = 9).

### Behavioral Analysis

The number of recognized trials and the reaction times for these trials were each analyzed using a *t*-test.

### fMRI Analysis

Data preprocessing and analysis was performed using the Statistical Parametric Mapping program, version 8 (SPM8; Ashburner et al., 2012) in MATLAB 2013. Data was motion corrected, normalized into MNI space (resampled to 3 × 3 × 3 mm) and smoothed with an 8 mm FWHM Gaussian kernel. A first-level GLM analysis was conducted using the canonical hemodynamic response function (HRF) and a 128 Hz high pass filter to remove slow drift. The six estimated motion parameters (three translations and three rotations) for each TR were included as nuisance regressors in the analysis. As each trial composed several stimuli in sequence (image onset, fixation and object appearance), the dispersion and derivative components of the HRF were not included in the model. Second-level independent samples *t*-tests were used to determine group effects. Given our *a-priori* hypotheses with regards to localization of activity, the effect of *context* was first assessed through hypothesis-driven ROI analyses (ROI definition described below) of the PHC and the RSC cortices (uncorrected *p*-value threshold = 0.01; voxel threshold = 10). Contrasts were performed using only recognized trials. Exploratory analyses of whole-brain activity were also performed to probe effect outside these two cortices (uncorrected *p*-value threshold = 0.005; voxel threshold = 10). The PHC ROI was generated using the AAL atlas (Tzourio-Mazoyer et al., 2002), which isolates the entire parahippocampal region. The coordinates of the significant activity levels were then visually inspected to determine whether they fell within the PHC. The RSC cortex was defined according to Brodmann Areas (BA 29 and 30) using the labels provided with MRIcron (Rorden and Brett, 2000). ROI masks were created using the parahippocampal region from AAL atlas, which were then applied to the second-level T maps in SPM8.

Beta values were extracted from both ROI and exploratory analyses by defining a roughly spherical boundary around the peak voxel. Only voxels with thresholds at the t-statistic were included (maximum 39 voxels per value). In the ROI analyses, beta value boundaries were constrained by the limits of the ROI.

## Results

### Behavioral Findings

The recognition rate (i.e., response “I recognize the object”) of objects presented in congruent context was significantly higher (71%, SD: 14%) than the recognition of objects presented in neutral context (53%, SD: 15%). According to our stimulus validation (see the “Materials and Methods” section), this difference is essentially due to the congruity of the contexts. The *t*-test showed that the effect of congruity was significant (*t*_(14)_ = 9.847, *p* < 0.001). Subjects reported being unable to recognize 16% (SD: 8%) of the objects appearing in congruent scenes and 29% (SD: 12%) of the objects appearing in neutral scenes.

### fMRI

**Figure 2:**
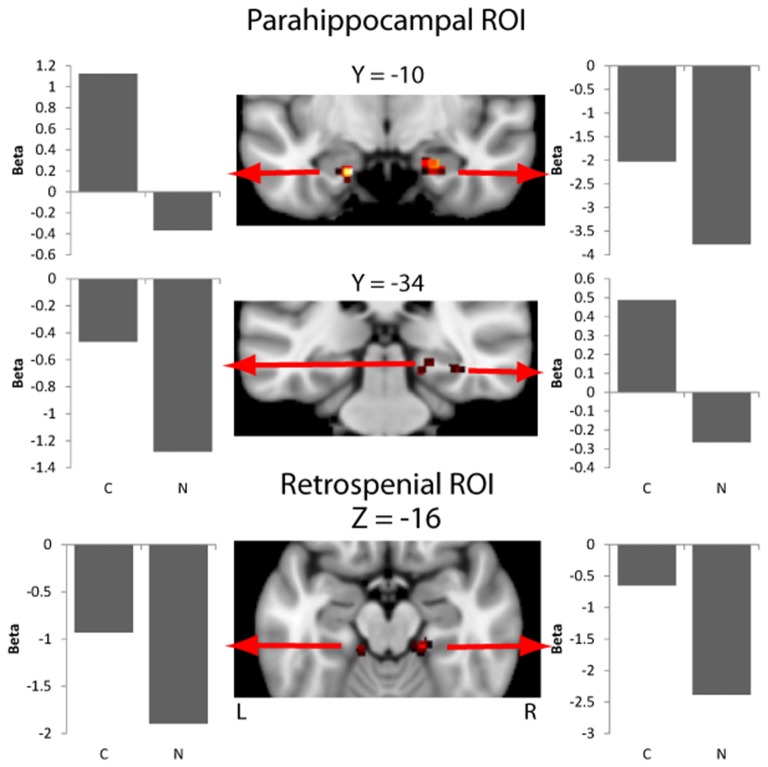
Activity in the parahippocampal cortex (PHC) and retrosplenial cortex (RSC) masks for the contrast of context effect (Congruent > Neutral). Beta values for each experimental condition are extracted from an ROI including of 11 voxels centered on the peak voxel (nine in plane plus one above and below the peak). Beta values are the model parameters generated by SPM that represent the amplitude of the effect for the condition being plotted.

Overall activity patterns for *Congruent* (C > N) stimuli that were recognized are reported in Table 1. *Congruent* stimuli elicited activity in the PHC, particularly in the right hemisphere. The PHC showed increased activity levels in both the ROI and exploratory analyses. RSC activity was also found and was more bilaterally distributed. Several other cortical areas were active in the exploratory (Figure 2) analyses, including the inferior parietal lobe (IPL), the precuneus and the fusiform gyrus, all in the right hemisphere. The largest cluster of activity was found in the cerebellum.

**Table 1:** Outcomes of functional magnetic resonance imaging (fMRI) contrasts for separate comparisons of context during recognition responses.

Peak *t* value	Voxels	*x*	*y*	*z*	Hemisphere	
**Parahippocampal ROI**
*Congruent > Neutral*
4.18	81	24	−10	−22	Right
5.87	41	−16	−10	−26	Left
3.34	15	22	−34	−12	Right
4.46	14	36	−34	−14	Right
**Retrosplenial ROI**
*Congruent > Neutral*
3.86	73	20	−34	−16	Right
3.19		16	−44	−20	Right
3.32	45	−12	−34	−16	Left
2.82		−14	−40	−24	Left
**Peak *t* value**	**Voxels**	***x***	***y***	***z***	**Hemisphere**	**Region**
**Exploratory analyses**
*Congruent > Neutral*
5.06	123	10	−20	−38	Right	Medulla
4.35		26	−34	−38	Right	Cerebellar tonsil
4.23		16	−28	−44	Right	Medulla
4.18	74	24	−10	−22	Right	Parahippocampal gyrus
4.69	53	44	−40	28	Right	Inferior parietal lobule
4.65	44	40	−38	−14	Right	Fusiform gyrus
5.87	40	−16	−10	−26	Left	Parahippocampal gyrus
3.86	21	20	−34	−16	Right	Culmen
3.19		16	−44	−20	Right	Culmen
3.75	19	32	−72	34	Right	Precuneus
3.85	18	22	−32	2	Right	Thalamus
3.96	14	24	−58	30	Right	Precuneus

## Discussion

The influence of scenes on the recognition of objects was examined in this study by making objects appearing in scenes that were either congruent with the object or neutral. PHC and RSC activity emerged when objects were viewed in congruent scenes; however, activity was also observed in the right parietal lobe, fusiform gyrus and cerebellar regions. These findings help specify the role of PHC and RSC regarding the object-context relationship, and indicate that different brain regions are employed when context helps to identify an object.

Our findings compliment the current state of knowledge, the PHC activity is particular to place-information of object during recognition while episodic memory is employed via the RSC when viewing congruent contexts to give the context meaning. These two brain areas are more activated by scenes than by objects (Epstein and Kanwisher, 1998; Epstein and Higgins, 2006) preferentially respond to scenes relative to faces or single objects. As the present study demonstrates, spatial information processed through the PHC is crucial for object identification: objects may be difficult to recognize partially because of the angle they are viewed from and having information about the position of an object in space may provide enough information to elicit its recognition. For example, an object in front of a desktop computer will likely be a keyboard. Our findings are further substantiated by the spatial layout hypothesis which states that the PHC processes information about the shape of space embodied in layout-defining scene features (Epstein and Kanwisher, 1998).

In the well-known Bar’s model of object recognition, the PHC and RSC mediate the familiar associations of information at two levels of abstraction (Bar, 2004; Bar et al., 2008). The PHC was found to be activated by spatial contextual information, including places, associated to specific objects (Bar and Aminoff, 2003). Spatial contextual information is processed quickly and provides representations that facilitate and fasten object recognition they are associated with by allowing predictions about what could appear in the scene (Fenske et al., 2006; Gronau et al., 2008). According to Bar (2009), these predictions are made before object is recognized, even when the scene is perceived simultaneously, because they are computed from low-level information that are quickly conveyed and processed. In the present study, the associative representations activated by the scenes were already accessible when object was perceived. This may have bolstered the effect of scene, although it has been shown in previous studies on event-related potentials that presenting the scene before or simultaneously has not an important effect on scene influence of object processing (Ganis and Kutas, 2003).

The present PHC activities is consistent with other evidences indicating that this brain structure plays a critical in the integration of objects within their scenes. For instance, in episodic memory tasks, the PHC was shown to be more activated for objects that were perceived in scenes where they had been initially studied (Hayes et al., 2007). However, other studies may have contradictory results such as in Rémy et al. (2014), where the PHC was more activated for incongruent than congruent scene-objects. This effect was, however, more likely related to the processing of incongruity than to the beneficial influence of scene on the recognition of the object.

The associations mediated within the RSC are more general and amodal than those mediated in the PHC. Objects that are related to spatial or nonspatial contexts activate more strongly the RSC than objects with no specific context (Bar and Aminoff, 2003). Based on other studies, the RSC activity may be unrelated to the processing of objects and be alternatively involved in a larger network mediating the spatial aspects of scenes (Epstein et al., 2007; Harel et al., 2013). While the present studies cannot specify the exact role played by the RSC, it nevertheless shows that it participates to the influence that scenes exert on the recognition of objects.

The exploratory analyses showed activity in the IPL and fusiform gyrus, both of which may also contribute to context meaning. The IPL has been associated with autobiographical memory and its activity when viewing congruent contexts suggests that past experiences with similar contexts are being accessed (Berryhill et al., 2007). The IPL has also been shown to play a crucial role in spatial processing and recognition of salient information, and it may help apply past memories to interpret presented stimuli (Battelli et al., 2007; Husain and Nachev, 2007; Singh-Curry and Husain, 2009). Likewise, the fusiform gyrus may also facilitate application of contextual memories to object recognition. Although it is best known for facial processing, previous studies suggest that it also facilitates top-down processing of contextual information and differently engages when viewing visible compared with obscured objects (Haynes et al., 2005; Fenske et al., 2006; Gutchess et al., 2006).

In summary, three key findings emerged: PHC activity appears to be specific to object identification, while activity in the RSC, IPL and fusiform gyrus may facilitate the recall of contextual meanings when viewing congruent scenes.

## Author Contributions

MBB is the main supervisor. He funded the project, designed the experiment, supervised the data collection and data analyses and significantly contributed to the writing. DC has collected data, analyzed the data and wrote the article. CH has significantly helped with the study design and data analyses.

## Conflict of Interest Statement

The authors declare that the research was conducted in the absence of any commercial or financial relationships that could be construed as a potential conflict of interest.
